# Analysis of Alterations in Intestinal Flora in Chinese Elderly with Cardiovascular Disease and Its Association with Trimethylamine

**DOI:** 10.3390/nu16121864

**Published:** 2024-06-14

**Authors:** Yannan He, Song Chen, Yuling Xue, Han Lu, Ziteng Li, Xianxian Jia, Yibing Ning, Qingbin Yuan, Shijie Wang

**Affiliations:** 1 College of Food and Biology, Hebei University of Science and Technology, Shijiazhuang 050018, China; tina7088@yeah.net (Y.H.); cs961113@163.com (S.C.); jingmaoluhan@126.com (H.L.); lztliziteng11@163.com (Z.L.); 2 Junlebao Dairy Group Co., Ltd., Shijiazhuang 050221, China; xueyuling@jlbry.com (Y.X.); yibingning@gmail.com (Y.N.); yuanqingbin@jlbry.cn (Q.Y.); 3 Institute of Basic Medicine, Hebei Medical University, Shijiazhuang 050017, China; hbydjiaxianxian@126.com

**Keywords:** trimethylamine (TMA), intestinal bacteria, elderly, cardiovascular disease (CVD), 16S rRNA sequencing, real-time fluorescence quantitative polymerase chain reaction (qPCR)

## Abstract

To investigate the changes in the intestinal flora in the Chinese elderly with cardiovascular disease (CVD) and its correlation with the metabolism of trimethylamine (TMA), the intestinal flora composition of elderly individuals with CVD and healthy elderly individuals was analyzed using 16S rRNA sequencing, the TMA levels in the feces of elderly were detected using headspace–gas chromatography (HS-GC), and four kinds of characterized TMA-producing intestinal bacteria in the elderly were quantified using real-time fluorescence quantitative polymerase chain reaction (qPCR). The results showed that *Firmicutes*, *Actinobacteria*, *Proteobacteria*, *Bacteroidetes*, and *Verrucomicrobia* are the dominant microorganisms of the intestinal flora in the Chinese elderly. And there were significant differences in the intestinal bacteria composition between healthy elderly individuals and those with CVD, accompanied by a notable difference in the TMA content. The richness and diversity of the intestinal flora in the elderly with CVD were higher than those in the healthy elderly. Correlation analysis indicated that certain significantly different intestinal flora were associated with the TMA levels. Our findings showed a significant difference in TMA-producing intestinal flora between healthy elderly individuals and those with CVD. The TMA levels were found to be positively and significantly correlated with *Klebsiella pneumoniae*, suggesting that this bacterium is closely linked to the production of TMA in the elderly gut. This may have implications for the development and progression of CVD in the elderly population.

## 1. Introduction

Cardiovascular disease (CVD) is a kind of disease that affects the heart and blood vessels, such as hypertension, coronary heart disease, heart failure, and stroke [1]. CVD poses a significant risk to the health of the elderly, making it a primary concern for their overall well-being. As the global population ages and the exposure to CVD risk factors increases among the elderly, CVD has become a prevalent disease in this population [2,3,4]. 

In the medical field, trimethylamine (TMA) is a risk marker for diseases, and its oxide TMAO is closely related to the occurrence and development of metabolic diseases such as CVD, chronic kidney disease, and diabetes. Choline, L-carnitine, and betaine are essential nutrients that the body obtains through diet. These nutrients are converted to (TMA) by the metabolism of the intestinal flora [5]. TMA in the gut is highly susceptible to absorption into the bloodstream by intestinal epithelial cells. Once in the bloodstream, TMA can have cytotoxic effects on cardiomyocytes, making it a potential toxin and cardiovascular risk marker [6]. Furthermore, TMA in the blood is transported to the liver through the portal circulation, where it is oxidized to trimethylamine-N-oxide (TMAO) by flavin monooxygenase. Clinical studies have shown that high levels of TMAO in the blood are closely related to the occurrence and development of cardiovascular disease (CVD) [7,8].

The gastrointestinal tract is the largest micro-ecosystem in the human body, and the gut flora plays a crucial role as a hidden “metabolic organ” with various physiological functions, such as facilitating sugar metabolism and protein hydrolysis, stimulating the immune system, and promoting innate immunity to pathogens and the regulation of mucosal barriers [9,10]. Additionally, the gut flora produces various metabolites, such as short-chain fatty acids, choline metabolites, bile acids, and uremic toxins, which can be transported to different organs through the bloodstream and affect human health [11]. Studies have revealed that the gut flora of individuals with CVD is significantly different from that of healthy individuals, with alterations in the composition and proportions of specific gut bacteria [12,13,14].

Therefore, this study investigated the differences in TMA content and changes in gut microbiota diversity between healthy elderly individuals and those with CVD in Shijiazhuang, Hebei Province, China, and explored the correlations between characteristic gut bacteria and TMA metabolism, as well as the relationship between the gut microbiota and cardiovascular diseases. This study aims to provide theoretical support for the rational control of TMA levels in the elderly population and the prevention of cardiovascular diseases in the elderly through the gut microbiota.

## 2. Materials and Methods

### 2.1. Volunteer Recruitment

The inclusion criteria for the study of elderly volunteers (aged 60 years or older) were as follows: participants were willing to cooperate with the study and provide stool samples. Volunteers were excluded if they had consumed probiotic or prebiotic products within the three months prior to sample collection. Individuals with unhealthy lifestyle habits, such as smoking and excessive alcohol consumption within the past three years, or those with severe drug dependencies, were not eligible. Additionally, individuals with major health issues, such as liver disease, acute or chronic pancreatitis, infectious diseases, or a history of major gastrointestinal surgeries, were also excluded from the study.

Twenty-five elderly volunteers were recruited in the Hebei region, we collected the fecal samples from them, and a follow-up questionnaire was completed at the time of sample collection. The questionnaire included information on participants’ weight, sleep patterns, exercise habits, dietary intake, and medication use. No dietary or lifestyle interventions were made during the course of the study.

### 2.2. Fecal Material

Fecal samples collected from the volunteers were delivered to the laboratory within 2 h at 4 °C for tube splitting, a portion of the sample was taken for TMA detection immediately after being thoroughly homogenized, and the remaining portion was frozen at −80 °C for subsequent 16S rRNA sequencing. No toilets were used in the collection of the feces to prevent contamination of the samples. The detailed information of the elderly sample is as follows (Table 1).

### 2.3. Extraction and Detection of TMA

Fecal samples collected from the volunteers were delivered to the laboratory at 4 °C and the pre-treatment was completed within 1 h. TMA was extracted by the following procedure: Homogenize the fecal sample, then take 1 g of the homogenized feces and add 4 mL of 0.06 mol/L sulfuric acid. Mix the sample well by shaking, then centrifuge it at 13,000× *g* for 10 min and aspirate the supernatant. Repeat this process twice more for the TMA in the feces’ residue and combine the supernatants from the three extractions into a single container. Mix the combined supernatants thoroughly. Add 5 mL of the supernatant to each of the two headspace vials (which are placed on ice), and add 5 mL of 5 mol/L, 20 mol/L sodium hydroxide solutions to the respective vials, then tightly close the vial caps. Finally, place the samples in the HS-GC instrument for detection.

The standard solutions were prepared as follows: Weigh 0.09 g of trimethylamine hydrochloride accurately and dissolve it in 0.06 mol/L sulfuric acid solution, then make up the volume to 80 mL to prepare a 696 mg/L TMA standard stock solution. Dilute the trimethylamine standard stock solution with 0.06 mol/L sulfuric acid to obtain a trimethylamine standard reserve solution with a concentration of 69.6 mg/L. Further dilute the trimethylamine standard reserve solution with 0.06 mol/L sulfuric acid to prepare a trimethylamine standard-use solution with a concentration of 6.96 mg/L, 3.48 mg/L, 1.74 mg/L, 0.87 mg/L, 0.435 mg/L, and 0.174 mg/L.

For the headspace instrument, the temperature of the heating chamber was 80 °C, the temperature of the quantification loop was 90 °C, the temperature of the transfer line was 110 °C, and the equilibration time was 40 min. Agilent 8890 gas chromatograph was used for the analysis of TMA. A 30 m × 0.32 mm CP-Volamine column with 5 μm thickness (Agilent, Santa Clara, CA, USA) was fitted to GC to separate TMA. The injection temperature was 200 °C in the splitting mode, and the shunt ratio was 10:1. The flow rate of the high-purity nitrogen carrier gas was 1.5 mL/min. The oven temperature was programmed at successive temperature and time points as follows: 50 °C for 6 min, increased to 240 °C at a rate of 3 °C/min, and held at 240 °C for 4 min. The temperature of the detector was 250 °C. The tailing blowing gas (nitrogen) flow rate was 30 mL/min, the hydrogen flow rate was 30 mL/min, and the air flow rate was 300 mL/min. HS-GC injection volume was 1 mL; all the data were calculated automatically by GC8890 chromatography workstation software (Agilent OpenLab version 3.5).

### 2.4. 16S rRNA Sequencing and Bioinformatic Analysis

The genomic DNA of each sample was extracted, and the DNA quality was detected on 1% agarose gels. Variable regions V3-V4 of bacterial *16S rRNA* gene were amplified with degenerate PCR primers, F338 (5′-ACTCCTACGGAGGGGGGGCAG-3′) and R806 (5′-GGACTACHVGGGTWTCTAAT-3′). PCR was performed by taking 30 ng of DNA samples of acceptable quality and the corresponding primers. The PCR amplification products were purified using Agencourt AMPure XP beads and dissolved in elution buffer. Fragment ranges and concentrations of the libraries were measured using an Agilent 2100 Bioanalyzer (Agilent, Santa Clara, CA, USA), and sequenced only if they met the qualification criteria. To generate Operational Taxonomic Unit (OTU) sequences, duplicated sequences were removed using QIIME2 software (version 2019.4), and clustering was performed at 100% similarity using the DADA2 method. The resulting unique sequences were collectively identified as OTUs.

The relative abundance of each species was calculated using OTUs, describing the gut microbiota composition of the elderly population at the phylum level, genus level, and enterotype level. Genera with an average relative abundance greater than 0.5% in the gut microbiota OTU results were selected as the dominant genera to explore widely distributed gut bacteria in the elderly population. α-diversity and β-diversity analyses were performed, along with species-level differences using the linear discriminant analysis effect size (LEfSe) method to investigate the diversity and differences in the bacterial composition of the gut microbiota among different groups of elderly individuals. This was performed to explore the gut microbiota diversity and differences in bacterial composition among various elderly populations.

### 2.5. Fecal Sample DNA Extraction and qPCR

Extraction of fecal flora DNA: The collected feces were homogenized and mixed, 0.2 g was taken into a 1.5 mL sterile centrifuge tube, and the DNA in the feces was extracted with the TIANamp Stool DNA Kit (TIANGEN BIOTECH, Beijing, China), and the extracted DNA was placed in the refrigerator at −20 °C for storage.

The primer sequences were utilized for the quantitative detection of 4 characterized enterobacteria species that produce TMA, including *Klebsiella pneumoniae*, *Clostridium sporogenes*, *Escherichia fergusonii*, and *Desulfovibrio desulfuricans* (Table 2). The plasmid expression vectors were constructed, and the copy number conversion was performed to establish the real-time fluorescence quantitative PCR standard curves for these four enterobacteria.

An ordinary PCR was carried out using a 12 µL reaction system for PCR amplification, which consisted of 6 µL of 2 × ES Taq MasterMix, 0.35 µL each of the upstream and downstream primers (5 µmol/L), 2 µL of DNA template, and deionized water to a final volume of 12 µL. The PCR conditions were as follows: an initial denaturation step at 95 °C for 3 min, followed by 40 cycles of denaturation at 95 °C for 30 s, annealing at 58 °C for 20 s, and extension at 72 °C for 30 s. After the cycling process, a final extension step was performed at 72 °C for 5 min. Subsequently, 10 µL of the PCR products was taken and subjected to electrophoresis in 2% agarose gel and 1× TAE buffer for 25 min (120 V) to identify and visualize the PCR products.

The plasmid concentration was formulaically converted to copy number according to the following formula:

DNA (copy)=6.02×10^23 (copy/mol)×DNA amount (g)DNA length (bp)×660 g/bp

The qPCR was performed using a 20 μL reaction system, which consisted of 2 μL of sample DNA, 0.6 μL of each of the upstream and downstream primers (5 mol/L), and 10 μL of 2 × SYBR Green qPCR Master Mix. The system was then topped up to 20 μL with deionized water. The qPCR procedure is outlined in Table 3.

### 2.6. Statistical Analysis

Statistical analysis and graph creation of the data were conducted using GraphPad. Data that did not follow a normal distribution were analyzed using non-parametric tests, while data that followed a normal distribution were analyzed using parametric tests, and *p* < 0.05 was considered significant. In addition, the Wilcoxon Test (for 2 groups) or the Kruskal–Wallis Test (for >2 groups) was utilized to analyze differences in microbial diversity among distinct groups. The Chao1 index and Shannon index were used to illustrate the α-diversity differences between heathy elderly and elderly with CVD, and differences in microbial composition between heathy elderly and elderly with CVD (β-diversity) were described using partial least squares discriminant analysis (PLS-DA) based on Unifrac distances. The top 10 species with the highest abundance in the intestinal flora were selected and a group-wise key species difference analysis was performed to further explore whether there were significant differences in the gut microbiota of elderly individuals in different groups at various taxonomic levels. And then the linear discriminant analysis effect size (LEfSe) was used for identifying the dominant bacteria in the gut microbiota of elderly individuals in different groups. The R packages (RStudio version 3.4.1) ggally package(version 2.2.1), corrplot package(version 0.92), and GraphPad Prism (version 9.5) were used to calculate and display the indicators.

## 3. Results and Discussion

### 3.1. Analysis of Fecal TMA Content in Elderly Adults

**Figure 1:**
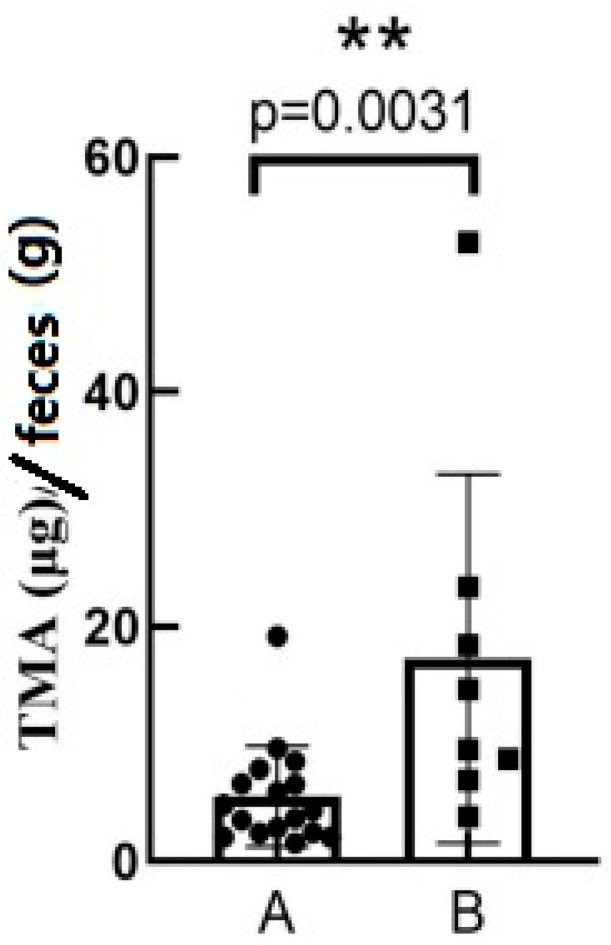
The concentration of TMA in the fecal samples of healthy elderly and elderly with CVD. Group A is a group of healthy elderly and group B is a group of elderly with CVD. ** denotes a significant level of *p* < 0.01.

Based on the results of the questionnaire survey (Table 1), the elders were divided into healthy elderly (group A) and elderly with CVD (group B). The measurement of TMA in 25 elderly fecal samples showed a significant difference between the feces’ TMA content of the healthy elderly population and that of the elderly population with CVD (Figure 1). The concentration of TMA in the CVD elderly was higher than that in the healthy elderly.

TMA precursors, such as choline and L-carnitine, consumed through diet are converted into TMA by the intestinal flora in the colon [17]. TMA in the colon is easily absorbed by colonic epithelial cells and enters the bloodstream, in the blood; TMA has been shown to be cytotoxic to cardiac cells and is considered as a risk marker for CVD [6,18]. Studies have shown that the TMA content in the blood of patients with CVD is negatively correlated with their glomerular filtration rate, and a decreased glomerular filtration rate is an independent risk factor for CVD [19]. Furthermore, TMA in the blood is oxidized into TMAO by FMOs in the liver, which promotes inflammation, affects cholesterol metabolism, and participates in thrombosis formation, leading to the occurrence and development of atherosclerosis (AS), which is the pathological basis of CVD [20]. Clinical studies have also shown that high levels of TMAO in the blood are closely related to the occurrence and development of CVD [21,22]. Currently, most studies focus on the relationship between TMAO and human diseases, with more common measurements of TMAO in the blood, and fewer measurements of TMA in feces and blood [5,23]. However, TMA in the blood is absorbed by colonic epithelial cells, and the absorption degree by these cells may vary among individuals. Moreover, TMAO is influenced by the enzyme activity of FMOs in the liver, and the expression and activity of FMOs may differ among individuals. Since research has shown that TMA in the blood is also related to CVD, it is necessary to measure TMA directly from the source to explore the relationship between TMA and diseases. This study found that the TMA content in the fecal samples from the CVD elderly individuals was higher than that of the healthy elderly individuals, and previous studies have also shown that the level of TMAO in the blood of heart failure patients is higher than that in healthy individuals [24].

### 3.2. Intestinal Flora Composition in the Elderly

**Figure 2:**
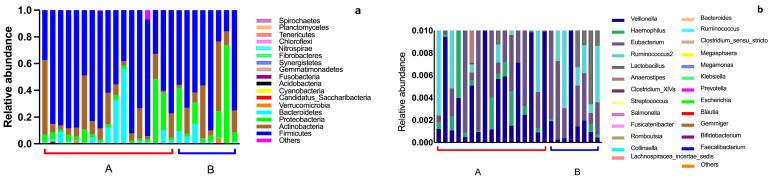
Basic microbial composition of 25 fecal samples at the phylum level (**a**) and the dominant genera (**b**). A represent healthy elderly, B represent elderly with CVD.

Based on *16S rRNA* gene sequencing technology, the composition of the intestinal flora in 25 elderly individuals was analyzed. The results showed that the intestinal flora of the elderly is mainly composed of *Firmicutes*, *Actinobacteria*, *Proteobacteria*, *Bacteroidetes*, and *Verrucomicrobia* (Figure 2a). These five phyla are also commonly found in the adult human gut microbiome. Among them, *Firmicutes* played a dominant role in the gut microbiota of most elderly. The relative abundance of *Proteobacteria* was higher in the elderly with CVD than the healthy elderly. In the analysis of the intestinal flora OTUs, we chose to focus on the genera with an average relative abundance exceeding 0.5% as the dominant taxa to investigate the prevalent intestinal flora found in the elderly population. In the intestinal flora of the elderly population, a total of 25 dominant genera were identified, including *Faecalibacterium*, *Bifidobacterium*, *Gemmiger*, *Blautia*, *Escherichia*, *Prevotella*, *Klebsiella*, *Megamonas*, *Megasphaera*, *Clostridium_sensu_stricto*, *Ruminococcus*, *Bacteroides*, *Lachnospiracea incertae sedis*, *Collinsella*, *Romboutsia*, *Fusicatenibacter*, *Salmonella*, *Streptococcus*, *Clostridium XlVa*, *Anaerostipes*, *Lactobacillus*, *Ruminococcus*, *Eubacterium*, *Haemophilus*, and *Veillonella* (Figure 2b).

Generally, there are significant differences in the composition and structure of the intestinal flora between the elderly and adults. Studies have shown that in populations aged 65 years and older, the community structure of the intestinal flora changes, and the abundances of *Bacteroidetes* and *Clostridium difficile* increase [25]. It is worth noting that the structure and composition of the intestinal flora in elderly individuals can differ at different ages. Studies have shown that the diversity of the intestinal flora in centenarians is significantly lower compared to that in 70-year-old individuals [26]. Moreover, lifestyle and living conditions also affect the intestinal flora of the elderly. Research has shown that the diversity of the intestinal flora in community-dwelling elderly individuals is different from that in long-term care residents [27]. In conclusion, when studying the intestinal flora of the elderly population, various factors should be considered, such as including elderly samples from the same region, having appropriate age-group divisions for the included elderly individuals, and ensuring that the lifestyles and living conditions of the included elderly individuals are as consistent as possible.

Just as the intestinal flora participates in maintaining host health, changes in the intestinal flora composition (such as gut dysbiosis) may promote the occurrence and development of host diseases. Previous experiments involving the transplantation of fecal microbiota into germ-free animals have shown that the intestinal flora is closely related to the occurrence and development of host diseases, such as obesity-related diseases, liver diseases, inflammatory bowel diseases, and colorectal cancer. The intestinal flora composition and structure in the elderly are different from those in young people. In the elderly, the ability of the intestinal flora to undergo metabolic processes production and starch breakdown decreases, while protein degradation activity increases [10].

### 3.3. Intestinal Flora Diversity in the Elderly

**Figure 3:**
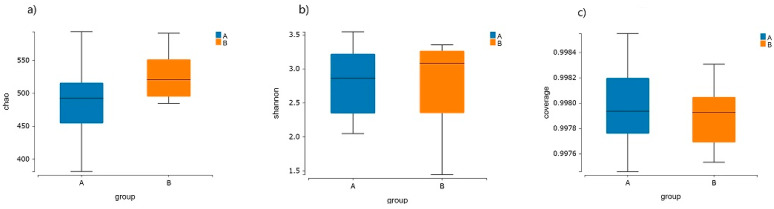
Graph of α-diversity index for the intestinal flora in different groups of elderly. Note: (**a**) The Chao index for the intestinal flora in different groups of elderly. (**b**) The Shannon index for the intestinal flora in different groups of elderly. (**c**) The box plot of the coverage indices for the intestinal flora in different groups of elderly. The Chao index reflects the abundance of intestinal flora among groups; a higher Chao index indicates more total species. The Shannon index is used to assess the homogeneity of species in the community; a higher index indicates higher homogeneity. The coverage index reflects whether the sample coverage is sufficient; the closer its value is to 1, the more likely it is that the two groups of microbial communities are sufficiently sequenced. Group A is a group of healthy elderly and group B is a group of elderly with CVD.

There was no significant difference (*p* > 0.05) in the α-diversity index of the intestinal flora between the healthy elderly group and the elderly group with CVD. However, the Chao index, which reflects the richness of the intestinal flora, and the Shannon index, which is part of the diversity index, were both lower in the healthy elderly group compared to the elderly group with CVD (Figure 3a,b). This suggests that there may be a decreasing trend in the richness and diversity of the intestinal flora in the healthy elderly relative to the elderly with CVD. The box plot of the coverage indices (Figure 3c) indicates that the values for both groups are greater than 0.99 and very close to 1. The difference is not statistically significant, suggesting that the sequencing can reflect the true conditions of the gut microbiota in the two groups.

**Figure 4:**
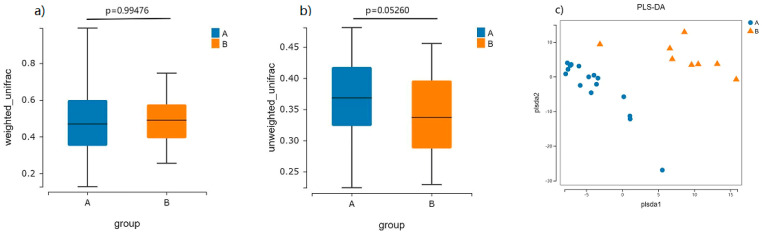
Graph of β-diversity index and PLS-DA for the intestinal flora in different groups of elderly. Note: (**a**) The Weighted_unifrac indices for the intestinal flora in different groups of elderly. (**b**) The Weighted_unifrac and Unweighted_unifrac indices for the intestinal flora in different groups of elderly. (**c**) The PLS-DA plots of the intestinal flora in the two groups of elderly samples. Beta diversity was used to assess the degree of difference in species communities between samples. The Unifrac algorithm including Weighted_unifrac and Unweighted_unifrac was used to compare the degree of difference in diversity of species between groups. Group A healthy elderly population and Group B CVD elderly population.

The results of the β-diversity analysis revealed that there were no statistically significant differences (*p* > 0.05) between the Weighted_unifrac and Unweighted_unifrac indices (Figure 4a,b). This suggests that there is no significant difference in the species composition and abundance of the intestinal flora between the healthy elderly and the elderly with CVD. From the PLS-DA plots of the two groups of elderly samples, it can be observed that the samples from the healthy elderly and the elderly with CVD were clearly separated on the horizontal and vertical axes, and the overall structure of the two groups of samples could be clearly distinguished (Figure 4c).

Due to the different species compositions and abundance structures of gut microbiota in the elderly population in different groups at the phylum, class, order, family, and genus taxonomic levels, this study selected the top 10 abundant species in the gut microbiota for intergroup key species difference analysis, further exploring whether there are significant differences in the gut microbiota at these taxonomic levels among different groups of elderly individuals.

The results showed that at the class level, the abundance of *Alphaproteobacteria* in the gut microbiota of the healthy elderly and CVD elderly were 0.106937% and 0.135762%, respectively (Table 4). Compared with the healthy elderly, the abundance of *Alphaproteobacteria* in the CVD elderly was significantly increased (*p* < 0.05). At the order level, the abundances of *Pasteurellales* in the gut microbiota of the healthy elderly and CVD elderly were 0.7909% and 0.01946%, respectively. Compared with the healthy elderly, the abundance of *Pasteurellales* in the CVD elderly was significantly decreased (*p* < 0.05).

**Figure 5:**
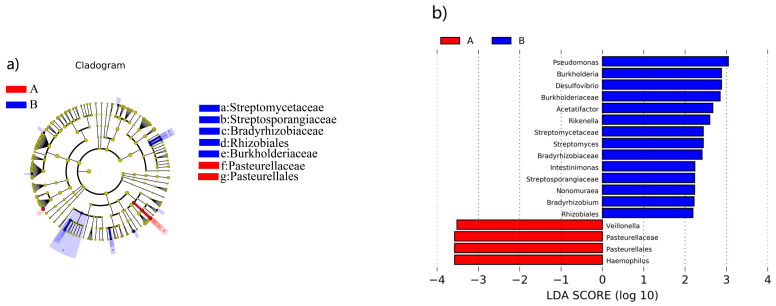
LEfSe analysis of microbial differences between healthy elderly and elderly with CVD. Note: (**a**) LEfSe clustering graph with red nodes representing microorganisms that play an important role in the healthy group, and graph with blue nodes representing microorganisms that play an important role in the CVD group; (**b**) LDA graph showing mainly statistically different biomarkers. A for the healthy elderly, and B for the elderly with CVD.

The results of the LEfSe differential discrimination analysis and key species significant difference analysis showed that there were seven significantly different bacteria in terms of abundance. Among these, *Pasteurellaceae* and *Pasteurellales* were found to be significantly higher in the intestines of healthy elderly individuals compared to those with CVD. On the other hand, *Streptomycetaceae*, *Streptosporangiaceae*, *Bradyrhizobiaceae*, *Rhizobiales*, and *Burkholderiaceae* were significantly more abundant in the intestines of the elderly with CVD compared to the healthy elderly (Figure 5a).

The LDA plot visualizes the differentiated species when the LDA value is >2, which means looking for enterobacteria with large differences. The LDA plot shows that in the samples from the healthy elderly population, *Veillonella*, *Pasteurellales*, *Pasteurellaceae*, and *Haemophilus* were significantly more likely to be associated with the healthy elderly population and greatly different from the elderly population with CVD. On the other hand, the elderly population with CVD had a significant association with *Pseudomonas*, *Burkholderia*, *Desulfovibrio*, *Burkholderiaceae*, *Acetatifactor*, *Rikenella*, *Streptomycetaceae*, *Streptomyces, Bradyrhizobiaceae, Bradyrhizobium, Intestinimonas, Streptosporangiaceae, Nonomuraea*, and *Streptomyces* (Figure 5b). Furthermore, *Streptosporangium*, *Nonomuraea*, *Bradyrhizobium*, and *Rhizobiales* showed significant differences compared to the healthy elderly population. When comparing the two differentially abundant bacteria obtained from the key species analysis with the significantly different species obtained by the LEfSe analysis, it was found that *Pasteurellales* could be the most promising key biomarker species to distinguish between the healthy elderly and elderly with CVD.

### 3.4. Analysis of the Characterized Enteric Bacteria That Produce TMA

**Figure 6:**
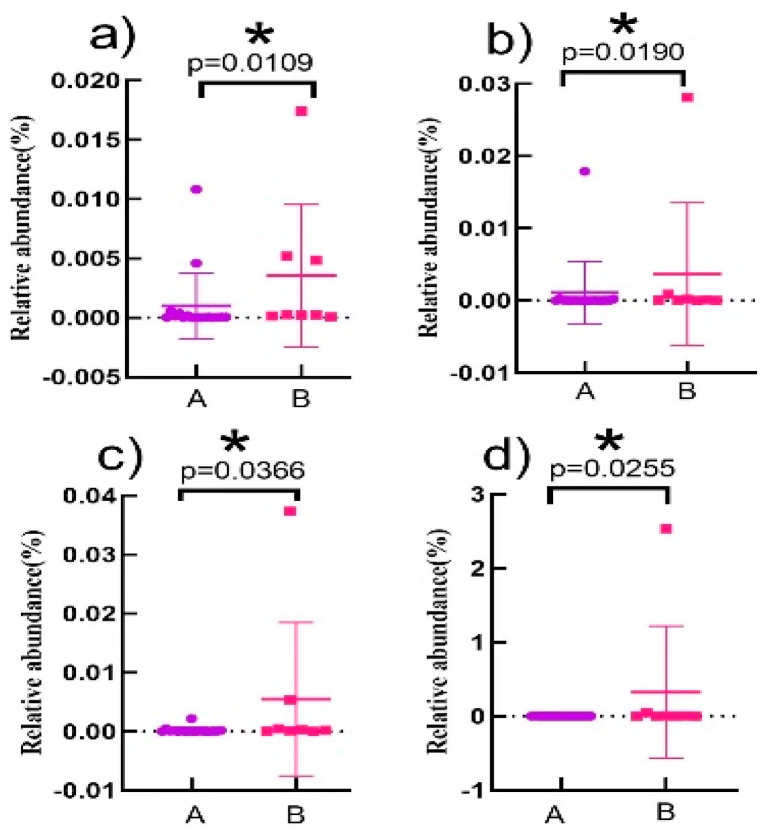
Abundance of four characteristic enteric bacteria in fecal samples from the elderly of different groups. (**a**–**d**) represent the differences between Klebsiella pneumoniae, Clostridium sporogenes, Escherichia Ferguson, and Desulfovibrio desulfuricans in healthy elderly and elderly with CVD, respectively. A represents the healthy elderly population and B represents the elderly population with CVD. * denotes a significant level of *p* < 0.05.

The relative abundance of four characteristic intestinal bacteria was significantly differentiated between the healthy and CVD elderly (Figure 6). And the relative abundance of Klebsiella pneumoniae (Figure 6a) ranged from 6.21 × 10^−5^% to 0.0174%, Clostridium sporogenes (Figure 6b) from 6.45 × 10^−6^% to 0.028%, Escherichia fergusonii (Figure 6c) from 1.51 × 10^−6^% to 0.037%, and Desulfovibrio desulfuricans (Figure 6d) from 1.87 × 10^−5^% to 2.53%. The statistical analysis indicated that the relative abundance of four characteristic intestinal bacteria was significantly lower in the healthy elderly than that in the elderly with CVD.

### 3.5. Correlation Analysis of Characteristic Intestinal Bacteria in the Elderly with TMA

**Figure 7:**
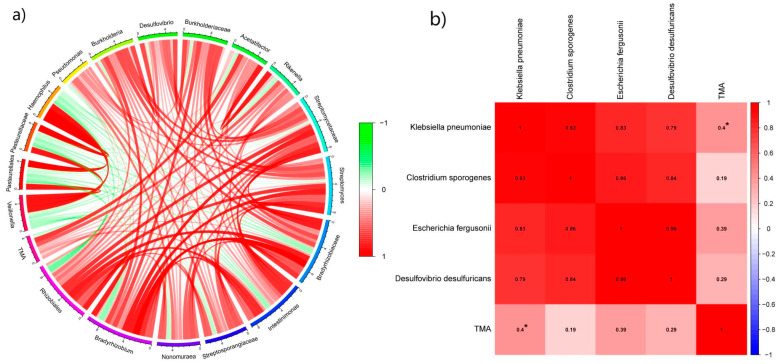
Correlation analysis of characteristic intestinal bacteria in the elderly with TMA. (**a**) Correlation analysis of 16SrRNA diversity analysis to obtain significantly different Enterobacteriaceae with TMA. (**b**) Correlation analysis of Enterobacteriaceae with TMA. * denotes a significant level of *p* < 0.05.

The differentially abundant bacteria identified through the LEfSe analysis were found to be associated with TMA, which showed a positive correlation with *Desulfovibrio*, *Acetatifactor*, *Rikenella*, *Streptomycetaceae*, *Streptomyces*, *Bradyrhizobiaceae*, *Intestinimonas*, *Streptosporangiaceae*, *Nonomuraea*, *Bradyrhizobium*, and *Rhizobiales* (Figure 7a). NCBI searches for the metabolic TMA-producing ability of these bacteria (mainly searching for the presence of the CutC and CntA/CntB genes) revealed that some strains of these bacteria have the potential to metabolize TMA. According to current reports, the microorganisms involved in TMA production are not limited to a single group but instead encompass microorganisms from various ecological niches. Most of these microorganisms are present at the species level [9]. As 16S rRNA can only be accurately analyzed to the level of microbial genera, its accuracy may be insufficient for identifying microorganisms at the species level [28,29]. Therefore, in this study, we utilized quantitative qPCR to quantify four characteristic enterobacteria species in the elderly gut that possess the ability to metabolize TMA. The primers for these four species were designed based on CutC sequences, which can provide accurate identification at the species level of the microorganisms [15]. The results of the correlation analysis between the four characterized enteric bacteria and TMA revealed that TMA exhibited a significant positive correlation with *Klebsiella pneumoniae* (Figure 7b). Furthermore, although positive correlations were observed between TMA and *Clostridium sporogenes*, *Escherichia fergusonii*, and *Desulfovibrio desulfuricans*, these correlations were not statistically significant. In conclusion, the feces’ TMA content in the elderly population is closely associated with the relative abundance of *Klebsiella pneumoniae* in their intestines. This finding suggests that the production of intestinal TMA in the elderly is more closely related to the presence of *Klebsiella pneumoniae* in their enteric bacteria.

The composition and structure of the intestinal flora are closely related to human health and diseases. Clinical studies have found that the oxidized product of TMA, TMAO, is closely related to the occurrence and development of CVD [30]. *Klebsiella pneumoniae*, *Clostridium sporogenes*, *Escherichia fergusonii*, and *Desulfovibrio desulfuricans* are the main intestinal bacteria that generate TMA. Accurate and rapid quantification of these bacteria is particularly important [15]. qPCR technology, based on DNA sequencing technology, is a widely used method known for its high specificity and strong sensitivity [31]. This technology has been successfully used for the quantitative analysis of specific bacterial populations and species in feces [32]. This technology has been used to quantitatively analyze microorganisms such as *Clostridium* spp., *Sutterella* spp., and *C. difficile* in the fecal samples of 18 healthy adults [33] and been used to detect the differences in the levels of *Bacteroidetes* and *Lactobacillus* between systemic lupus erythematosus patients and healthy individuals [34]. Jomehzadeh N employed qPCR to study the number of *lactobacillus* species in the gut of constipated children and healthy children, detecting seven major *lactobacillus* species in fecal samples, and the results of the study showed that the number of *lactobacillus* species in the patient group were significantly reduced compared to those in the healthy subjects [35]. These findings demonstrate that qPCR can be applied in the detection of fecal intestinal flora and has advantages such as rapidity, efficiency, and good repeatability. Currently, there are few reports on the qualitative and quantitative detection of TMA-producing intestinal flora in fecal samples at home and abroad. This study established a qPCR method for quantifying the abundance of four bacteria in fecal samples by designing specific primers for *Klebsiella pneumoniae*, *Clostridium sporogenes*, *Escherichia fergusonii*, and *Desulfovibrio desulfuricans*. The results demonstrated that this method has a good specificity and low detection limit, making it suitable for the rapid detection of gut microbiota in fecal samples.

*Klebsiella pneumoniae*, *Clostridium sporogenes*, *Escherichia fergusonii*, and *Desulfovibrio desulfuricans* are currently reported to exist in the human gut and be capable of producing TMA. Investigating the differences in these bacteria among individuals with different health conditions is essential for studying the correlation between the intestinal flora and human physiology and pathology. The results of this study showed that there is a significant difference in the relative abundance of *Klebsiella pneumoniae*, *Clostridium sporogenes*, *Escherichia fergusonii*, and *Desulfovibrio desulfuricans* between CVD elderly and healthy elderly individuals, which is consistent with current research reports [36,37]. The analysis of the relationship between characteristic intestinal flora and TMA revealed that the four specific intestinal bacteria have a positive correlation with the concentration of TMA. Furthermore, a significant positive correlation was observed among these four characteristic intestinal bacteria, suggesting that these four TMA-producing intestinal bacteria may have a mutually promotional effect on one another.

## 4. Conclusions

In this study, HS-GC technology was used to investigate the differences in TMA content between the healthy elderly and those with CVD in China. 16S rRNA sequencing was used to analyze the intestinal flora composition of these elderly individuals. Furthermore, qPCR technology was explored to quantify the characterized TMA-producing intestinal bacteria. The results showed that the dominant microorganisms of the intestinal flora of Chinese elderly are *Firmicutes*, *Actinobacteria*, *Proteobacteria*, *Bacteroidetes*, and *Verrucomicrobia*. There were significant differences in the concentration of TMA in the feces, and the composition and diversity of the intestinal flora between healthy elderly and those with CVD. The richness and diversity of the intestinal flora in the elderly with CVD were higher than those in the healthy elderly. The results of the qPCR and correlation analysis indicated that the TMA levels were found to be positively and significantly correlated with *Klebsiella pneumoniae*, suggesting that this bacterium is closely linked to the production of TMA in the elderly gut. This may have a significant impact on the development and prevention of CVD in the elderly.

## Figures and Tables

**Table 1:** Detailed information on elderly fecal samples.

	Gender	Age	Height (cm)	Weight (kg)	Physical Condition	Group
1	Female	65	160	51	CVD (diabetes, hypertension, hyperlipidemia and hyperglycemia)	B
2	Male	66	168	71	CVD (cerebral infarction)	B
3	Female	62	160	61	healthy	A
4	Male	63	165	70	CVD (hypertension)	B
5	Male	68	165	65	healthy	A
6	Female	68	155	60	CVD (cerebral infarction)	B
7	Female	66	156	60	CVD (hypertension)	B
8	Male	71	164	65	CVD (cardiopathy)	B
9	Female	60	150	60	CVD (hypertension, hyperglycemia)	B
10	Male	65	171	73	healthy	A
11	Male	73	165	65	healthy	A
12	Male	72	171	65	CVD (cardiopathy)	B
13	Female	65	162	65	healthy	A
14	Male	69	165	70	healthy	A
15	Female	61	159	60	healthy	A
16	Female	70	164	65	healthy	A
17	Male	66	173	60	healthy	A
18	Female	67	155	48	healthy	A
19	Male	65	160	65	healthy	A
20	Female	66	1.62	68	healthy	A
21	Female	70	155	60	healthy	A
22	Male	63	165	65	healthy	A
23	Female	60	155	60	healthy	A
24	Male	70	160	64	healthy	A
25	Male	60	159	64.5	healthy	A

**Table 2:** Species’ PCR-specific primers.

Target	Sequence (5′→3′)	Amplicon Length (bp)	References
Klebsiella pneumoniae	F-GATCTGACCTATCTGATTATGG R-TTGTGGAGCATCATCTTGAT	185	[9]
Clostridium sporogenes	F-TCGTGAAGCAGGAGTATGGG R-GTCAACACGTCCTATAGACATACC	460	[15]
Escherichia Ferguson	F-AGCGAACTGGGAGCGAAATA- R-TACGACCACGGTTGAGGACA	421	[15]
Desulfovibrio desulfuricans	F-CGTGTTGACCAGTACATGTA R-GCTGGTAACCTGCGAAGAA	163	[9]
Total bacteria	F-ACTCCTACGGGAGGCAGCAGT R-GTATTACCGCGGCTGCTGGCAC	190	[16]

**Table 3:** Real-time fluorescent quantitative PCR program.

Stage	Cycle	Temperature (°C)	Time (s)
Stage 1		95	300
Stage 2	40	95	15
		58	20
		72	40
Stage 3	Melting curve		

**Table 4:** Key difference species of gut microbiota in the elderly population.

Grape	Species Level	Species	*p* Value
A, B	class	*Alphaproteobacteria*	0.038635
	order	*Pasteurellales*	0.01208

## Data Availability

The original contributions presented in the study are included in the article, further inquiries can be directed to the corresponding author.
